# Decreased Activity of Blood Acid Sphingomyelinase in the Course of Multiple Myeloma

**DOI:** 10.3390/ijms20236048

**Published:** 2019-11-30

**Authors:** Marzena Wątek, Ewelina Piktel, Joanna Barankiewicz, Ewa Sierlecka, Sylwia Kościołek-Zgódka, Anna Chabowska, Łukasz Suprewicz, Przemysław Wolak, Bonita Durnaś, Robert Bucki, Ewa Lech-Marańda

**Affiliations:** 1 Institute of Hematology and Transfusion Medicine, Indiry Gandhi 14, 02-776 Warsaw, Poland; joanna.barankiewicz@gmail.com (J.B.); ewamaranda@wp.pl (E.L.-M.); 2 Department of Microbiology and Immunology, The Faculty of Medicine and Health Sciences of the Jan Kochanowski University in Kielce, Stefana Żeromskiego 5, 25-001 Kielce, Poland; przemyslaw.wolak@ujk.edu.pl (P.W.); Bonita.Durnas@onkol.kielce.pl (B.D.); buckirobert@gmail.com (R.B.); 3 Department of Medical Microbiology and Nanobiomedical Engineering, Medical University of Bialystok, Mickiewicza 2c, 15-222 Bialystok, Poland; ewelina.piktel@wp.pl (E.P.); lukaszsuprewicz@gmail.com (Ł.S.); 4 Holy Cross Cancer Center, Artwinskiego 4, 25-734 Kielce, Poland; ewa.baranska1@wp.pl (E.S.); sylwia.kosciolek.zgodka@gmail.com (S.K.-Z.); 5 Regional Blood Transfusion Center in Bialystok, 15-950 Bialystok, Poland; mchabowska@rckik.bialystok.pl; 6 Centre of Postgraduate Medical Education, Marymoncka 99/103, 01-813 Warsaw, Poland

**Keywords:** myeloma multiple, sphingolipids, acid sphingomyelinase, cell signaling

## Abstract

Acid sphingomyelinase (aSMase) is involved in the generation of metabolites that function as part of the sphingolipid signaling pathway. It catalyzes the breakdown of sphingomyelin into ceramide, a bioactive lipid that, among other roles, is involved in regulation of apoptosis. Dry drop blood test (DBS) and colorimetric 2-step enzymatic assay were used to assess the activity of human blood aSMase, beta-galactosidase, and beta-glucosidase, these enzymes are lysosomal hydrolases that catalyze the degradation of related sphingolipids, of sphingolipid signaling molecules. Blood was collected from a group of healthy volunteers and patients that were diagnosed with multiple myeloma (MM) in various stages of the disease. Additionally, activity of those enzymes in patients diagnosed with other hematological cancers was also assessed. We found that aSMase activity in the blood of patients with MM (at the time of diagnosis) was 305.43 pmol/spot*20 h, and this value was significantly lower (*p* < 0.030) compared to the healthy group 441.88 pmol/spot*20 h. Our collected data suggest a possible role of aSMase in pathogenesis of MM development.

## 1. Introduction

In multiple myeloma (MM), development of clonal plasmocyte populations defines the progress of the disease. Plasmocytes suppress the normal blood cell line, leading to immunosuppression and impairment of normal hematopoiesis. They also cause lytic bone lesions and impair renal function through several different mechanisms [1]. Understanding the biology of the tumor that leads to the MM progression is crucial for effective treatment [2]. It is known that the genetic predisposition, the inflammation accompanying the disease, and the abnormal immune response, are all involved in the development of MM. Initiating genomic disorders occur during the maturation of B cells, and clonal plasma cells occupy niches in the bone marrow (BM). The early stage of MM is called monoclonal gammopathy of undetermined significance (MGUS). More than 10% of bone marrow involvement is characterized by the next stage of MM termed as an asymptomatic MM. Damage to tissues (mostly bones and kidneys) defines a symptomatic disease that requires treatment. Aggressive plasmocyte clones often show complete inactivation of tumor suppressor genes, such as *TP53* [2]. Asymptomatic patients should not be treated unless they have end-organ damage. It is necessary to classify patients as having a high-risk or standard-risk disease. Qualification for the risk group is determined by genetic status of abnormal plasmocytes. Hypodiploidy or deletions (del) of chromosome 13 (del [13]), presence of t (4;14), t (14; 16), t (14; 20) translocations, or del (17p) are considered as high-risk factors. Overall, 25% of patients with symptomatic MM are classified as high-risk patients [3]. Interestingly, MGUS and MM are more frequent in patients with a congenital defect of sphingolipid metabolism [4].

**Figure 1:**
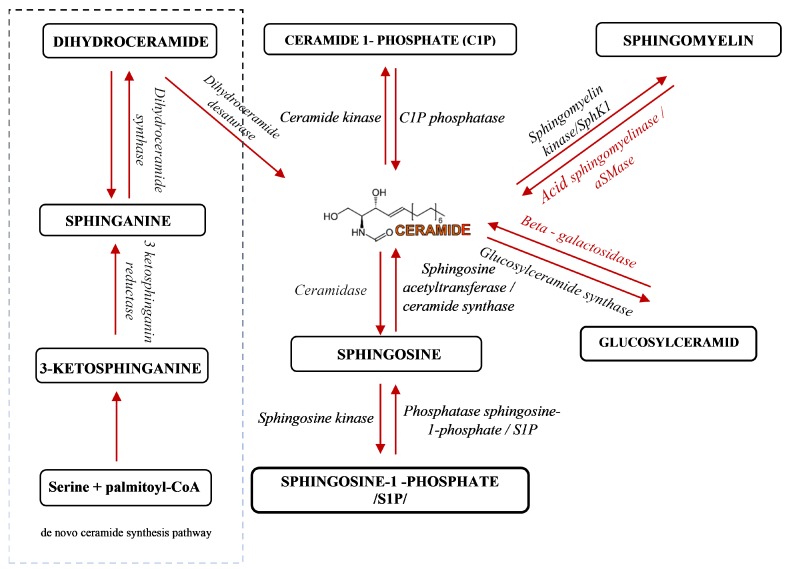
A schematic representation of sphingolipid metabolism [5]. Arrows indicate the direction of compounds synthesis.

**Figure 2:**
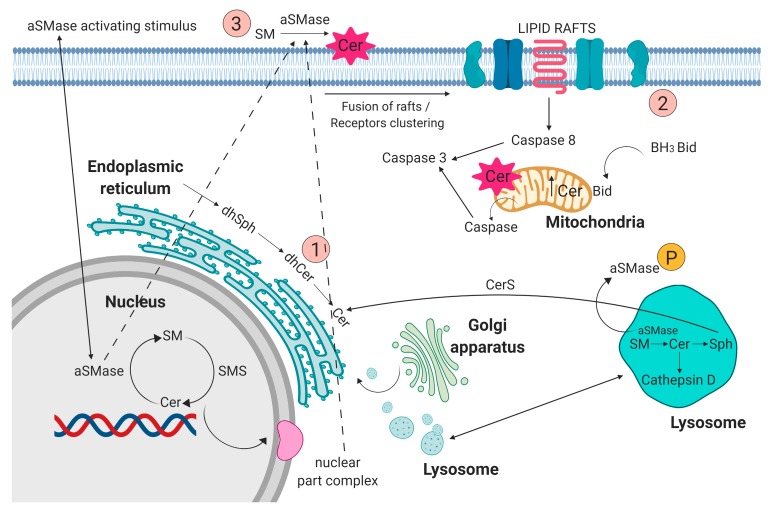
(**1**) Sphingolipids, after synthesis in the endoplasmic reticulum, nucleus, and Golgi apparatus, are transported to cell membrane. (**2**) Acid sphingomyelinase (aSMase) contributes to fusion of lipid rafts and formation of membrane microdomains. (**3**) aSMase acting on the plasma membrane contribute to ceramide production. Figure prepared using BioRENDER software.

Sphingolipids have been known for years as structural molecules of the cell membrane. They have been shown to be involved in many important cell-signaling processes and their production is governed by the different enzymes (Figure 1) [5]. After synthesis in the endoplasmic reticulum and Golgi apparatus, they are transported to the cell membrane (Figure 2 (1)). They participate in the regulation of numerous cellular processes. Sphingolipids regulate growth, differentiation, chemotactic motility, and control cell death (apoptosis). Sphingolipid molecules tend to self-assemble, forming microdomains within the cell membranes, called lipid rafts [6,7] (Figure 2 (2)). They are also compounds of various, often contradictory, biological properties. The main secondary messenger of the sphingomyelin (SM) signaling pathway occupying a central role in the sphingolipid metabolism is ceramide (Cer) (Figure 2 (3)) [6]. It forms as a result of sphingomyelin cell membrane hydrolysis, by sphingomyelinase enzymes, and its de novo synthesis starts from serine and palmitoyl-CoA. This reaction is catalyzed by the enzyme palmityl transferase and the product of its action is 3-ketosfinganine [8]. Ceramide induces apoptosis, whereas sphingosine-1-phosphate (S1P) plays a pivotal role in cell survival [9].

The sphingomyelin signaling pathway was described in lymphoid tissue, spleen, nerve tissue, lungs, liver, myocardium, skeletal muscle, and gastrointestinal mucosa [10,11]. Metabolic linking of sphingolipids through mutual interactions related to the action of enzymes: sphingosine kinases, S1P phosphatases, ceramide synthases, and ceramidases, are important in normal cell function and pathology of disease [9]. Abnormal sphingolipids metabolism is characteristic feature of Gaucher disease, and those patient have increased incidence of neoplasm based on population data [4]. Based on the results of studies regarding the incidence of neoplasms in the group of patients with Gaucher disease, an increased incidence of tumors in congenital sphingolipidosis has been observed. It seems that disorders of sphingolipid metabolism in this disease may constitute a model of cancer pathogenesis [12]. Clinical observations showed that in Gaucher disease, congenital disorders of sphingolipid metabolism [4] and damaged GBA1 enzyme activity [13] promoted the occurrence of malignant disease, in particular of MGUS, MM, and B-cell lymphomas, that are more frequent than in the healthy population [12], especially commonly described in GD1 (the most frequent Gaucher disease type I) [14,15]. Nair et al. suggest that the basis of both Gaucher disease-related gammopathies and some sporadic monoclonal gammopathies are the result of long-term immune activation by lysolipids (lysolipid GL1 and lysophosphatidylcholine). They show that substrate reduction improves gammopathy associated with Gaucher disease in mice [16]. In a group of 28 patients (19 men, 9 women) with congenital acid sphingomyelinase deficiency (ASMD), Niemann-Pick type A disease (NPD), Lidov et al. showed abnormal production of gammaglobulin in the form of polyclonal hypergammaglobulinemia in 6 patients (*n* = 6) and MGUS in 5 (*n* = 5) [17]. Enzyme acid sphingomyelinase (aSMase) is encoded by the sphingomyelin phosphodiesterase 1 (SMPD1) gene. It has been shown that the p.L302P mutation of the SMPD1 gene is a strong risk factor for Parkinson’s disease [18]. However, they have also been shown to play a significant role in many cancers, generally reducing the levels of proapoptotic lipid, ceramide, and/or increasing the levels of proliferative lipid, sphingosine-1-phosphate (S1P). It is noteworthy that recombinant human aSMasa (rhASM) has been produced for human use and is being evaluated as a NPD treatment. Thus, its use in cancer therapy is a near future in animal model research [19]. Lee et al. in 1982 showed the presence of cancer in 19 out of 20 examined subjects, and the most common cancer was MM [20]. Another congenital sphingolipidosis that has been shown to have oncogenic disorders related to the loss of aSMase is Niemann-Pick type A disease. aSMase hydrolyzes sphingomyelin, producing ceramides, but the aSMase protein targets remain largely unclear. Proteomic analyses based on mass spectrometry identified >100 proteins associated with aSMase-dependent, detergent-resistant membrane microdomains (lipid rafts). Over 60% of these proteins are palmitoylated, including synaptosomal-associated protein 23 kinases (SNAP23), Src-family kinases Tak and Lyn, and Ras and Rab families (superfamily of small GTPases). Inactivation of aSMase removes the presence of these proteins in the plasma membrane. Many of them in aSMase deficiency were trapped in the Golgi apparatus. It has been shown that aSMase is required for the transport of palmitoylated proteins (such as SNAP23 and Lyn) from the Golgi apparatus to the plasma membrane. Importantly, aSMase delivered extracellularly can regulate SNAP23 movement from the Golgi apparatus to the plasma membrane. Research suggests that aSMase, acting on the plasma membrane to produce ceramides (Figure 2), regulates the localization and translocation of palmitylated proteins [21]. Expression of the Met proto-oncogene (also called c-Met) is incorrectly upregulated in many human tumors, e.g., glioma. Activation of Met receptor tyrosine kinase (Met RTK) by a related ligand, hepatocyte growth factor (HGF), triggers mitogenesis and morphogenesis and is essential during embryonic development, cell migration, wound healing, and angiogenesis [22].

The evaluation of the enzyme activity of aSMase, beta galactosidase, and beta glucosidase using dry blood spot (DBS) test in patients with MM in different stages of the disease was carried out to assess the possible implications of those enzyme in MM development. Indeed, obtained data indicate a potential link between MM pathogenesis and sphingolipids metabolism.

## 2. Results

### 2.1. Clinical Characteristics of the Patient Group

The main characteristics of the patients are presented in Table 1. A group of 100 patients was examined, of which 49 were women and 52 were men.

Fourteen MM patients at the time of diagnosis were examined, 55 MM patients who were on treatment, 14 patients with other cancers, and 17 healthy volunteers. The group of patients with cancers other than MM included: patients with primary myelofibrosis (*n* = 3), marginal zone lymphoma (*n* = 4), chronic lymphocytic leukemia (*n* = 3), common B-cell Philadelphia chromosome–negative[B-Ph(-)] acute lymphoblastic leukemia (*n* = 2), and hairy cell leukemia (*n* = 2).

### 2.2. Outline of Beta Glucosidase and Beta Galactosidase Activity

**Figure 3:**
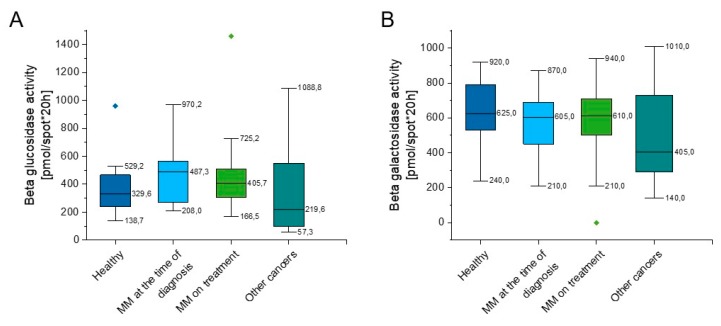
Activity of beta glucosidase (panel (**A**)) and beta galactosidase (panel (**B**)) in the blood of healthy subjects in the control group (*n* = 17), patients with MM at the time of diagnosis (*n* = 14), patients with MM during cytostatic treatment (*n* = 55), and patients with other hematological tumors (*n* = 14). Data are presented as median and 25–75% percentile of obtained results. Whiskers of each box mark the minimum and maximum values within the data set that fall within an acceptable range (±1.5 interquartile range [IQR]). Any value outside of this range is displayed as an individual point.

As indicated in Supplementary Table S1 and Figure 3, some slight increase of beta glucosidase activity in both groups of MM patients in comparison to healthy volunteers (*p* = 0.152, *p* = 0.311) was observed (Figure 3A), nevertheless this did not reach statistical significance. The beta galactosidase activity of the above groups was comparable (*p* = 0.462, *p* = 0.507); however, there was a lower activity of beta galactosidase in the group of hematological tumors other than myeloma when compared to the group of healthy people (*p* = 0.129) (Figure 3B).

### 2.3. Outline of Acid Sphingomyelinase (aSMase) Activity

**Figure 4:**
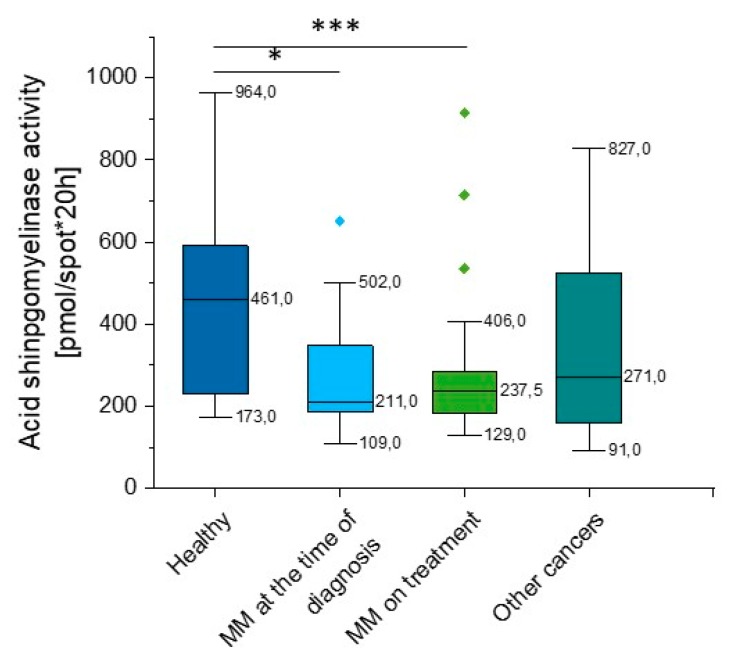
Activity of acidic sphingomyelinase (aSMase) in the blood of healthy subjects in the control group (*n* = 17), patients with MM at the time of diagnosis (*n* = 14), patients with MM during cytostatic treatment (*n* = 55), and patients with other hematological tumors (*n* = 14). * and *** indicate statistical significance (*p* < 0.05 and *p* < 0.001, respectively) comparing to healthy patients. Data are presented as median and 25–75% percentile of obtained results. Whiskers of each box mark the minimum and maximum values within the data set that fall within an acceptable range (±1.5 interquartile range [IQR]). Any value outside of this range is displayed as an individual point.

According to our data, there is a significant reduction in aSMase activity in MM patients when compared to healthy volunteers (*p* = 0.008). Importantly, this correlation was observed both at the time of diagnosis (*p* = 0.039) and in patients during treatment (*p* = 0.0035). We did not observe statistical significance in other hematological neoplasms, but the heterogeneity of the group should be emphasized (*p* = 0.119) (Supplementary Table S1, Figure 4).

**Figure 5:**
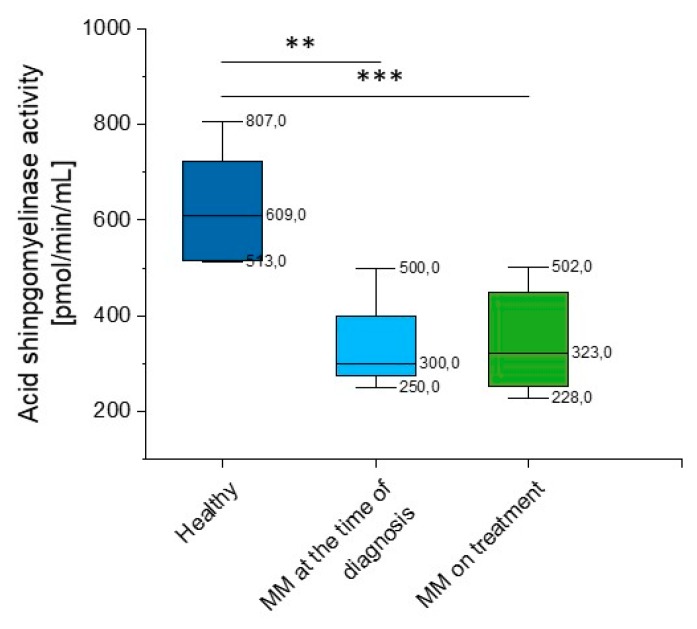
Activity of acidic sphingomyelinase (aSMase) in the blood of healthy subjects in the control group (*n* = 20), patients with MM at the time of diagnosis (*n* = 3), and patients with MM during cytostatic treatment (*n* = 7). ** and *** indicate statistical significance (*p* < 0.01, and *p* < 0.001, respectively) comparing to healthy patients. Data are presented as median and 25–75% percentile of obtained results. Whiskers of each box mark the minimum and maximum values within the data set that fall within an acceptable range (±1.5 interquartile range [IQR]).

Given the considerable variability of aSMase blood levels in MM patients detected using the DBS test, we performed additional analysis of acid sphingomyelinase activity using the colorimetric two-step enzymatic assay. The study confirmed previous results, a significant reduction in aSMase (*p* = 0.028 and *p* = 0.003 for MM patients at the time of diagnosis and on treatment, respectively) (Supplementary Table S2, Figure 5) was observed.

### 2.4. Outline of Ceramide, Sphingosine-1-Phosphate (S1P), Sphingosine (SFO), and Sphinganine (SFA) Concentration

In order to evaluate whether concentrations of related sphingolipids, including sphingomyelin species and ceramide, are altered in MM patients, we performed analysis of their levels in a representative group of patients and healthy subjects.

**Figure 6:**
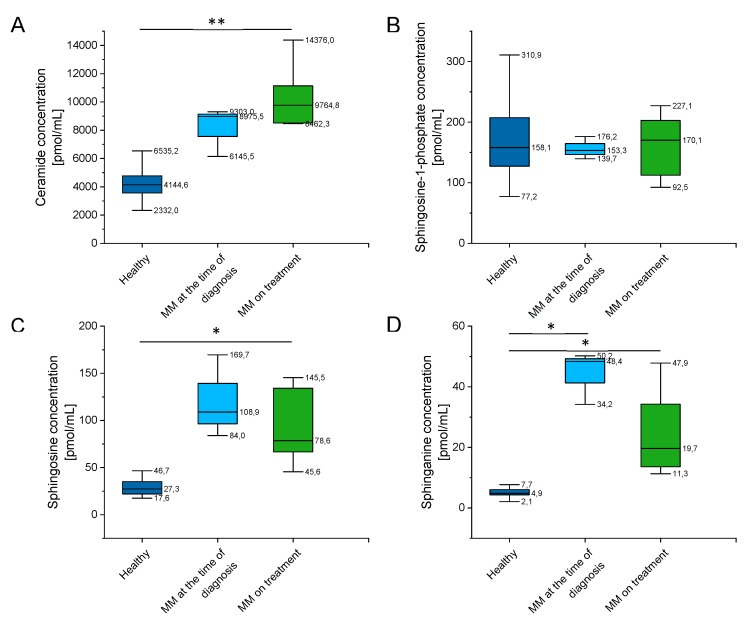
Concentration of ceramide (panel (**A**)), sphingosine-1-phosphate (S1P) (panel (**B**)), sphingosine (SFO) (panel (**C**)) and sphinganine (SFA) (panel (**D**)) in the blood of healthy subjects in the control group (*n* = 20), patients with MM at the time of diagnosis (*n* = 3), patients with MM during cytostatic treatment (*n* = 7). * and ** indicate statistical significance (*p* < 0.05, *p* < 0.01, respectively) comparing to healthy patients. Data are presented as median and 25–75% percentile of obtained results. Whiskers of each box mark the minimum and maximum values within the data set that fall within an acceptable range (±1.5 interquartile range [IQR]).

As indicated in Supplementary Table S3 and Figure 6, there were a significantly higher concentration of ceramide, sphingosine (SFO), and sphinganine (SFA) in MM patients when compared to healthy volunteers, and no significant alterations of sphingosine-1-phosphate (S1P) levels in this group. Additionally, some impact of chemotherapy treatment on their blood concentration is noted (particularly for sphingosine and sphinganine, *p* value below 0.001).

### 2.5. Outline Enzyme Activity Depending on the International Staging System (ISS) for Multiple Myeloma (MM)

**Figure 7:**
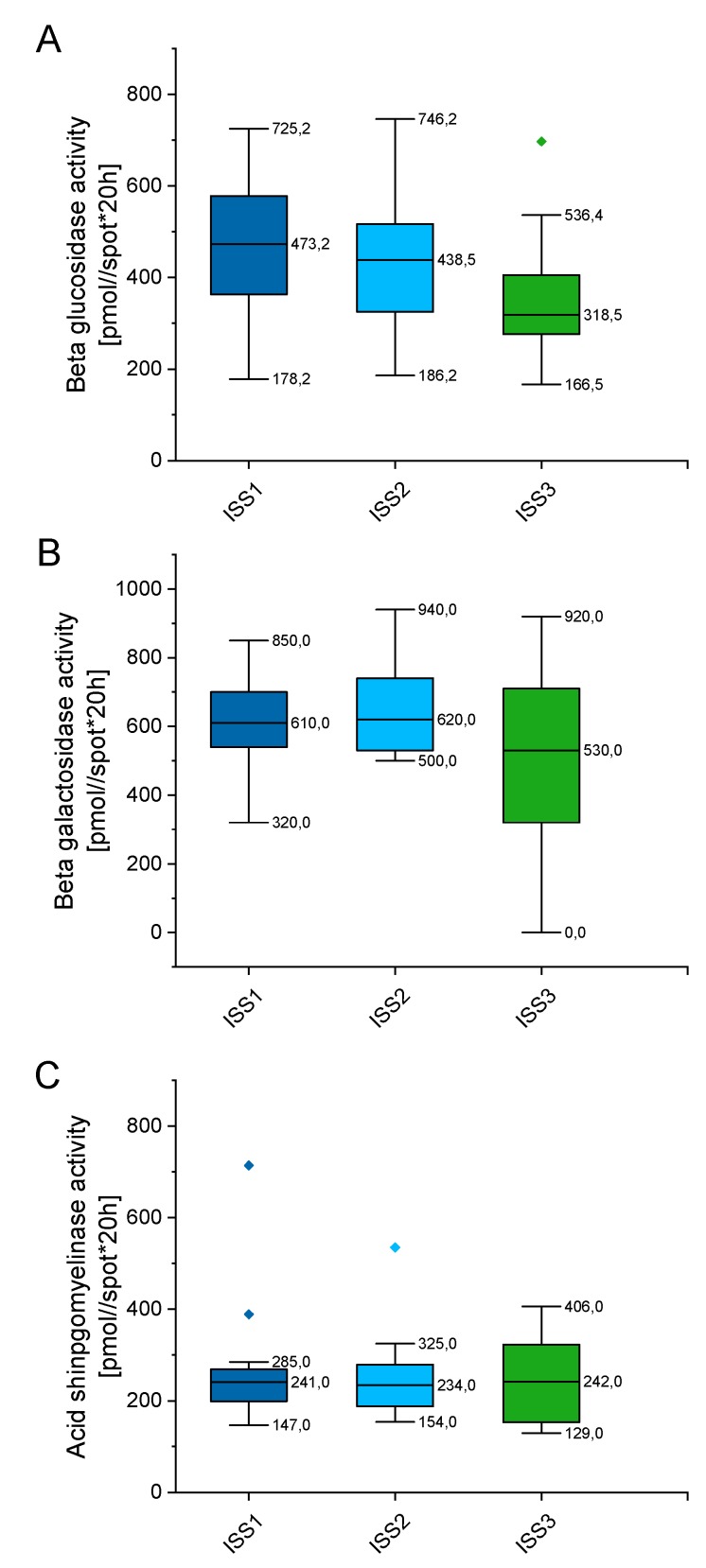
Concentration of beta glucosidase (panel (**A**)), beta galactosidase (panel (**B**)), and acid sphingomyelinase (panel (**C**)) in the blood of patients with MM depending on the stage of myeloma (international staging system; ISS). *n* (ISS1) = 19, *n* (ISS2) = 32, *n* (ISS3) = 18. Data are presented as median and 25–75% percentile of obtained results. Whiskers of each box mark the minimum and maximum values within the data set that fall within an acceptable range (±1.5 interquartile range [IQR]). Any value outside of this range is displayed as an individual point.

In order to check whether enzymes’ activity alter with disease progression, we classified tested patients by the international staging system (ISS), which prognosticates the severity of multiple myeloma (Supplementary Table S4). As indicated in Figure 7, the enzymes’ activity varies depending on the stage of myeloma, but these alterations did not reach statistical significance. When compared with patients classified as the ISS1 group, no statistical significance was recorded for the ISS2 and ISS3 groups of patients (*p* value ranging from 0.899 to 0.1169). Above might suggest that alterations in above enzymes, particularly aSMase, might be involved in the pathogenesis of MM, but do not impact further progression of disease.

## 3. Discussion

More than 40 years ago, the term apoptosis was introduced to describe programmed cell death. Apoptosis is still the subject of intense research. Key players of apoptosis at the molecular level have been identified, such as caspases, death receptors, and members of the Bcl-2 protein family. More than 20 years after this initial description, ceramide was described as a regulator of this phenomenon. Sphingolipids, bioactive components of cell membranes, are involved in numerous physiological functions. The major part of apoptosis among sphingolipids is ceramide. It affects the aggregation of the death receptor in the plasma membrane and the formation of pores in the mitochondria [23,24].

aSMase plays an important role in initiating ceramide-mediated cell signaling [25]. In cell membranes and membranes of intracellular complexes (Golgi complex, endoplasmic reticulum, or mitochondria), dynamic structures that constantly and quickly change their composition, sphingolipids, and cholesterol, build cell heterogenous regions of membrane domains/lipid rafts, composed of sphingolipids and cholesterol (Figure 2 (2)). These structures play an important role in many biological processes, including proliferative, informational, and apoptotic processes. Changes in the lipid composition of lipid rafts lead to the rearrangement or relaxation of the actin-membrane bonds. The balance of the recycling of storage organelles (late endosomes and lysosomes) is the decisive factor in regulating the expression levels of membrane proteins. The sorting of membranes that occurs in endosomes is important for the regulation of cell physiology [4]. Hydrolysis of sphingomyelin to ceramide with the participation of aSMase initiates membrane reorganization and facilitates the formation and coalescence of lipid microdomains [25].

In our study, we showed that the aSMase activity in the blood of MM patients is significantly lower than in that of the healthy population. It was previously shown that activation of aSMase leads to the accumulation of ceramide and induces apoptotic cell death in tumor cells [3]. Consequently, the aSMase deficiency that we have found in MM patients can lead to prolonged myeloma cell survival. The controversy of researchers on the participation of aSMase in cell death via Fas was assessed in the mouse model by Lin et al. They showed that aSMase affects cell death via Fas in some, but not all, tested tissues [26].

A bioactive messenger of the sphingolipid information pathway, ceramide has pro-apoptotic activity and inhibits tumor growth [12]. As a result of the degradation of ceramide by the enzyme ceramidase, sphingosine (Sph) is formed, followed by further metabolism sphingosine-1-phosphate (S1P), which affects carcinogenesis and regulates inflammation in many other processes. The broad spectrum of biological activity of the sphingolipid pathway compounds is due to the diversity of receptors associated with the sphingomyelin signaling pathway [4]. The pro-apoptotic function of ceramide also involves the activation of enzymes which are involved in cytoskeletal rearrangement, such as caspase 3 and 8 (Figure 2). The activity of the ceramide tumor suppressor is associated with the induction of tumor cell apoptosis and tumor growth slowing by transfer of apoptotic signals by T cells. Inhibition of ceramide production impairs interleukin-2 (IL-2) production and suppressed programmed cell death after T-cell induced receptor activation (TCR) [4].

Ceramide concentration in the examined group of patients with MM was significantly higher than in the blood of the control population. Some published data have shown that ceramide induction can protect some cancer cells from death. For example, ceramide C16 produced by ceramide synthase (CerS6) has been shown to play an important role in protecting head and neck squamous cell carcinomas (HNSCC) against apoptosis mediated by stress endoplasmic reticulum (ER) [27]. De novo ceramide synthesis generates ceramide molecules with varying amounts of carbon atoms (ranging from 14 to 26) long chain sphingosine (LCB) combined with a fatty acyl chain (with different biological roles in cancer cells probably due to different subcellular location and cell communication) [28].

MM is an incurable disease with a median survival time of six years [29] with treatment that involves the use of new drugs, such as lenalidomide and bortezomib or high-dose chemotherapy with autologous stem cell transplantation [30,31]. Many studies point to the sphingolipid pathway as an important regulator affecting cancerous cell growth. S1PR signaling has been shown to play a key role in cell survival in many hematological cancers (not only myeloma) and as a factor governing drug resistance. The impairment of the apoptotic process is confirmed by experience with the S1P inhibitor, fingolimod (FTY720). S1P, opposite to ceramide, plays a key role in cell survival [9]. In MM cell lines cultures, S1P increases cell proliferation, but treatment with FTY720P decreases the proliferation by reducing the expression of target genes.

Knocking down S1P5R results in a decreased expression of the cell survival genes; it does not affect the expression of drug resistance genes [32]. In the studies on T cell acute lymphoblastic leukemia (T-ALL), it was shown that the accumulation of ceramide produced in the process of sphingomyelin hydrolysis, as well as de novo synthesis, plays an important role in information on death transmitted to leukemic cells, also affects antigen regulation CD95 (APO-1 / Fas). Treatments used in patients with ALL (doxorubicin, daunorubicin, cytarabine, fludarabine) induce cell apoptosis by both inclusion in cellular DNA and stimulation of ceramide production [12]. Several authors suggest that the drugs such as fludarabine, vincristine (VCR), and cytarabine (AraC) induce apoptosis by inducing ceramide production [33]. In our earlier studies in patients with acute myelogenous leukemia, we showed a profile of sphingolipids in the blood and bone marrow of the subjects being different. More precisely, in acute myeloid leukemia (AML) patients, the average SFO, SFA, and CER concentration in blood plasma was significantly higher compared to the control group, when blood plasma S1P concentration was significantly lower. At the same time, the CER/S1P ratio in AML patients was about 54% higher compared to the control group. Interestingly, the average concentration of S1P in blood plasma was higher compared to its concentration in plasma collected from bone marrow. Perhaps we still underestimate the role that sphingolipids play in the development of hematological malignancies and their influence on the disease therapy [12]. The lipid profile obtained in the current observation is consistent with the results obtained in our previous study in patients with leukemia.

It was also shown that in MM cells, the activity of sphingosine 1 kinase (SphK1), a negative ceramide accumulation regulator, was significantly higher compared to normal peripheral blood mononuclear cells. Silencing the activity of SphK1 through the polyphenol epigallocatechin-3-O-gallate of green tea (EGCG) intensified the apoptotic effect of the aSMase (in opposite to the action of SphK1) on apoptosis activator [34]. Targeted 67LR receptor activation of aSMase induces lipid rafting disorders and inhibits RTK activation in MM cells. EGCG induces aSMase translocation into the cell membrane and phosphorylation of Cδ protein kinase (PKCδ) at Ser664, which is necessary for signaling through aSMase/ceramide and 67LR. Moreover, it was shown in the Lin et al. experiment, that the grouping of lipid rafts and the apoptotic death of MM cells is mediated by the EGCG, a 67LR receptor inducer, by the activation of protein kinase C and aSMase. These results explain the new cell death pathway triggered by EGCG for MM cell death [20]. Activation of aSMase by targeting 67-kDa-encoded laminin receptors (67LR) induces disruption of the lipid rafts and inhibits RTK activation in MM cells [3]. In CD 138+ cells in newly diagnosed MM, one third of the genes involved in sphingolipid metabolism, including the sphingosine 2 kinase (SK2) and S1P genes, were significantly different than those of normal individuals, which shows SK2 dysregulation in myeloma. The SK2 inhibitor ABC294640 has been shown to induce apoptotic death, as demonstrated by annexin V staining, poly (ADP-ribose) polymerase (PARP), and caspase 9 activation of both primary myeloma cells and myeloma cell lines [35].

Our results are in agreement with previous data describing the development of myeloma in patients with congenital sphingolipidoses, and suggest that imbalances in the formation of ceramide, SFO, SFA, S1P, end enzymes activity, such as aSMase, may affect the development of the disease and change as a result of its treatment. The results encourage us to further analyses, including investigation to assess potential SPL gene mutations within a group of patients suffering from MM. Such studies might help assess the carrier state, therefore a genetic risk factor for MM development.

## 4. Materials and Methods

### 4.1. Acquiring Blood Samples

We used blood collected from a group of 100 patients from the Hematology Clinic of the Holly Cross Oncology Center in Kielce, hospitalized between January 2016 and January 2018. Blood was collected from patients diagnosed with MM, including patients subjected to chemotherapy (at least 3 weeks after last dose of chemotherapy). Diagnosis of MM was based on WHO criteria 2016 [36]. Additionally, we also collected blood from healthy volunteers (control group). Experiments aiming on assessment of shingolipid concentrations were performed using serum collected from 10 MM patients and 20 healthy volunteers from the Institute of Hematology and Transfusion Medicine in Warsaw.

The experiments were performed according to the principles in the Declaration of Helsinki. The research was approved on 30.01.2015 by the Bioethical Commission of the Jan Kochanowski University in Kielce No.3/2015. Patients’ written consent was obtained to submit the study.

### 4.2. Blood Drop Test

The blood drop test DBS from Sanofi Genzyme (Cambridge, MA, USA) was used to evaluate blood activity of beta galactosidase, beta glucosidase, and aSMase. Diagnosis of congenital sphingolipidosis was performed using mass spectrometry in the Metabolic Laboratory, Dept. Of Pediatrics, Hamburg University Medical Center, Martinistr 52, 20246 Hamburg, Germany, as previously described [37,38].

### 4.3. Colorimetric Evaluation of aSMase Activity

Colorimetric 2-step enzymatic assay (Cambridge, United Kingdom, cat no. ab252889) was used in order to confirm the results of the DBS test. For this purpose, a representative group of MM patients (*n* = 10; at the point of diagnosis: *n* = 3; MM on treatment: *n* = 7) was utilized and aSMase activity was compared with group of healthy volunteers (*n* = 20). Sample acid sphingomyelinase activity was calculated from choline standard curve and expressed as (pmol/min/mL).

### 4.4. Assessment of Phospholipids Serum Levels

The levels of ceramide, sphingosine (SFO), sphinganine (SFA), and sphingosine-1-phosphate (S1P) were determined as previously described [39]. Briefly, lipids were extracted from 250 μL of plasma in the presence of internal standards (10 pmol C17-sphingosine and 30 pmol C17-S1P, Avanti Polar Lipids). An aliquot of the lipid extract was transferred to a fresh tube with pre-added 40 pmol N-palmitoyl-Derythro-sphingosine (C17 base; a kind gift of Dr. Z. Szulc, Medical University of South Carolina) as an internal standard, and then subjected to alkaline hydrolysis to deacylate ceramide to SFO. The amount of S1P was determined indirectly after dephosphorylation to SFO, with the use of alkaline phosphatase (bovine intestinal mucosa, Fluka). Free SFO, dephosphorylated sphingoid bases, and SFO released from ceramide were then converted to their o-phthalaldehyde derivatives and analyzed using a HPLC system equipped with a fluorescence detector and C18 reversed-phase column (Varian Inc., OmniSpher 5, Palo Alto, United States; 4.6 × 150 mm). The isocratic eluent composition of acetonitrile (Merck, Darmstadt, Germany):water (9:1, *v*/*v*) and a flow rate of 1 mL/min were used.

### 4.5. Statistical Analysis

Differences between groups were assessed using the non-parrametric Mann-Whitney-Wilcoxon test, with *p* < 0,05 as the level of significance.

## 5. Conclusions

aSMase activity in blood of patients diagnosed with MM is significantly reduced regardless of the stage of disease. Even if additional studies are required, it is likely that evaluation of aSMase activity might have an important diagnostic value. These results require further analysis and better understanding of their meaning in the context of MM pathogenesis. Overall, these results suggest that S1P/ceramide ration and S1P and ceramide–mediated signaling have an effect on the proliferation and apoptosis of MM cells, therefore it might govern their drug resistance.

## Figures and Tables

**Table 1:** Clinical characteristics of the patient groups.

Group	Patients	Median Age	M:F
Healthy	17	52.58 (29–79)	12:5
MM at the time of diagnosis	14	65.5 (42–86)	8:6
MM on treatment	55	65.13 (42–88)	24:31
Other cancers *	14	54.28 (28–76)	9:5

* primary myelofibrosis (*n* = 3), marginal zone lymphoma (*n* = 4), chronic lymphocytic leukemia(*n* = 3), common B-cell Philadelphia chromosome–negative[B-Ph(−)] acute lymphoblastic leukemia (*n* = 2), and hairy cell leukemia (*n* = 2). MM = multiple myeloma.

## Supplementary Materials

Supplementary materials can be found at https://www.mdpi.com/1422-0067/20/23/6048/s1.

## Abbreviations

SM	Sphingomyeline
aSMase	Acid sphingomyelinase
Cer	Ceramide
Sph	Sphingosine
CerS	Ceramide synthase
