# Supplementary material for: Decreased Activity of Blood Acid Sphingomyelinase in the Course of Multiple Myeloma
Source: Int J Mol Sci. 2019 Nov 30;20(23):6048. doi: 10.3390/ijms20236048 (PMC6929010; doi:10.3390/ijms20236048)
Supplement: Supplementary file 1 [file ijms-20-06048-s001.pdf]

# Supplementary Materials

**Supplementary Table S1.** Activity of beta glucosidase, beta galactosidase and acid sphingomyelinase in blood of tested patients. Results are presented as (pmol/spot*20 h). *p* value indicates statistical differences when compared to health subjects.

| | Beta Glucosidase | | Beta Galactosidase | | Acid Sphingomyelinase | |
|---|---|---|---|---|---|---|
| | Activity (pmol/spot*20 h) | *p* value | Activity (pmol/spot*20 h) | *p* value | Activity (pmol/spot*20 h) | *p*-Value |
| Reference values | **200–2000** | | **0,5–3,2** | | **200–3500** | |
| Healthy | **375,69 ± 194,84** (138,74–959,88) | | **0,63 ± 0,19** (0,24–0,92) | | **441,88 ± 221,42** (173–964) | |
| Myeloma at the time of diagnosis | **469,09 ± 216,64** (207,97–970,17) | $p = 0.152$ | **0,58 ± 0,17** (0,36–0,87) | $p = 0.462$ | **305,43 ± 153,55** (156–751) | $p = 0.039$ |
| Myeloma on treatment | **436,9 ± 240,88** (166,52–746,17) | $p = 0.311$ | **0,59 ± 0,18** (0,21–0,94) | $p = 0.507$ | **262,62 ± 136,89** (129–914) | $p = 0.003$ |
| Other cancers | **347,63 ± 310,01** (57,3–1088,85) | $p = 0.349$ | **0,50 ± 0,28** (0,14–0,94) | $p = 0.129$ | **346,64 ± 243,69** (91–827) | $p = 0.119$ |

* primary myelofibrosis (*n* = 3), marginal zone lymphoma (*n* = 4), chronic lymphocytic leukemia (*n* = 3), common B Ph (−) acute lymphoblastic leukemia (*n* = 2), and hairy cell leukemia (*n* = 2)

**Supplementary Table S2.** Activity of acid sphingomyelinase in blood of tested patients. Results are presented as (pmol/min/mL). *p* value indicates statistical differences when compared to health subjects.

| **Control Group ($n$ = 20)** | **Study Group ($n$ = 10)** | **(pmol/min/mL)** | ***p*-Value** |
|---|---|---|---|
| 629 ± 119 | MM at the time of diagnosis ($n$ = 3) | 394 ± 99 | $p$ = 0.02819 |
| | MM on treatment ($n$ = 7) | 341 ± 105 | $p$ = 0.00341 |

**Supplementary Table S3.** Concentration of ceramide, S1P, SFO and SFA in blood of tested patients. Results are presented as pmol/mL. *p* value indicates statistical differences when compared to health subjects.

| Compound | Control Group ($n = 20$) | Study Group ($n = 10$) | (pmol/min/mL) | *p*-Value |
|---|---|---|---|---|
| Ceramide | 4281,52 ± 1148,3 | MM at the time of diagnosis ($n = 3$) | 8141,35 ± 1736,2 | $p = 0.00935$ |
| | | MM on treatment ($n = 7$) | 9764,8 ± 2026,43 | $p = 0.000507$ |
| S1P | 167,95 ± 54,99 | MM at the time of diagnosis ($n = 3$) | 156,35 ± 18,446 | $p = 0.97502$ |
| | | MM on treatment ($n = 7$) | 159,35 ± 51,62 | $p = 0.71256$ |
| SFO | 28,752 ± 8,03 | MM at the time of diagnosis ($n = 3$) | 120,88 ± 44,099 | $p = 0.05532$ |
| | | MM on treatment ($n = 7$) | 93,929 ± 36,77 | $p = 0.000598$ |
| SFA | 5,17 ± 1,39 | MM at the time of diagnosis ($n = 3$) | 44,26 ± 8,75 | $p = 0.00531$ |
| | | MM on treatment ($n = 7$) | 23,72 ± 4,95 | $p = 0.00505$ |

**Supplementary Table S4.** Clinical manifestations of the group of patients and monoclonal paraprotein concentrations.

| | | Durie-Salmon Staging System | ISS | Patients No. |
|---|---|---|---|---|
| MM at the time of diagnosis | IgA kappa | III A | 2 | 1 |
| | IgG kappa | I A | 1 | 1 |
| | IgG kappa | I A | 2 | 2 |
| | IgG kappa | II A | 2 | 2 |
| | IgG kappa | III A | 2 | 4 |
| | IgG lambda | III A | 1 | 1 |
| | IgG lambda | III A | 2 | 1 |
| | IgG lambda | III A | 3 | 1 |
| | IgA lambda | III A | 3 | 1 |

| | | Durie-Salmon Staging System | ISS | Patients No. |
|---|---|---|---|---|
| MM on treatment | IgA kappa | I B | 3 | 1 |
| | IgA kappa | III A | 1 | 3 |
| | IgA kappa | III A | 2 | 2 |
| | IgA lambda | III A | 3 | 2 |
| | LCD kappa | III A | 1 | 1 |
| | LCD lambda | III A | 2 | 2 |
| | LCD lambda | III A | 3 | 1 |
| | IgG kappa | II A | 1 | 2 |
| | IgG kappa | II A | 2 | 3 |
| | IgG kappa | III A | 1 | 6 |
| | IgG kappa | III A | 2 | 9 |
| | IgG kappa | III A | 3 | 4 |
| | IgG kappa | III B | 3 | 1 |
| | IgG lambda | III B | 2 | 1 |
| | IgG lambda | III B | 3 | 3 |
| | IgG lambda | III A | 1 | 4 |
| | IgG lambda | III A | 2 | 3 |
| | IgG lambda | III A | 3 | 1 |
| | Plasmacytoma solitare | IIIA | 2 | 2 |
| | Plasmacytoma solitare | IIIA | 3 | 2 |
| | Plasmacytoma solitare | I A | 1 | 1 |
